# Supplementary material for: hapln1a+ cells guide coronary growth during heart morphogenesis and regeneration
Source: Nat Commun. 2023 Jun 13;14:3505. doi: 10.1038/s41467-023-39323-6 (PMC10264374; doi:10.1038/s41467-023-39323-6)
Supplement: Supplementary file 1 — Supplementary Information [file 41467_2023_39323_MOESM1_ESM.pdf]

Supplementary Figures and Legends

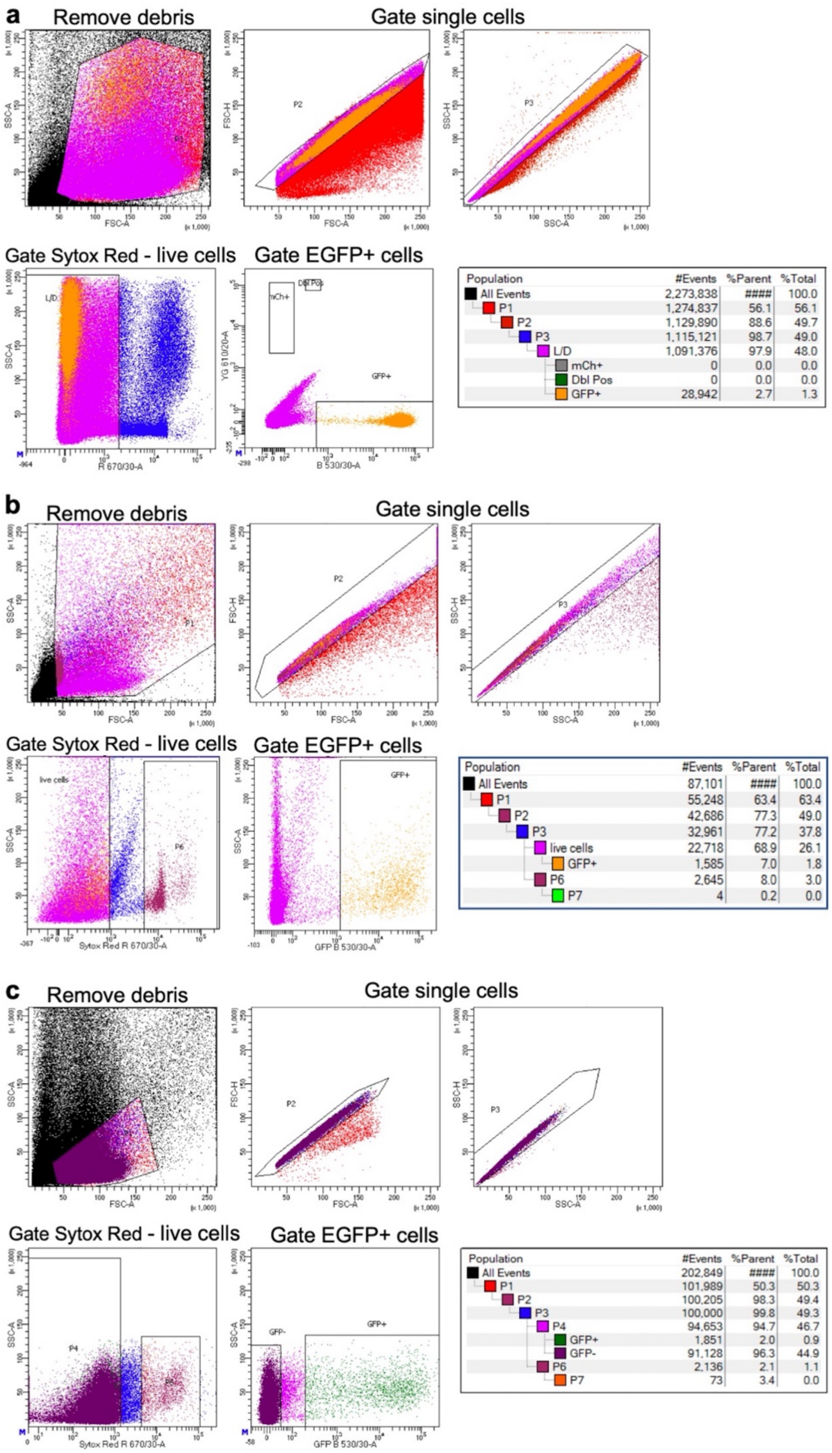

Supplementary Fig. 1: Flow cytometry plots showing the single-cell sorting process for EGFP$^+$ cells from juvenile hearts of *tcf21:nucEGFP* (**a**) and *hapln1a:EGFP* fish (**b**) at 7 wpf, and EGFP$^+$ cells from the hearts of adult *hapln1a:EGFP* fish at 7 dpa (**c**). BD

FACSDiva v9.0 software was used for analysis. Total events were visualized with a FSC-A Vs SSC scatter plot from which the main Population 1 (P1) was gated to discard cell debris. Exclusion of double event was performed using a FSC-A Vs FSC-W scatter plot from which population 2 (P2) was gated. GFP and DAPI was assessed using a two-parameter scatter plot.

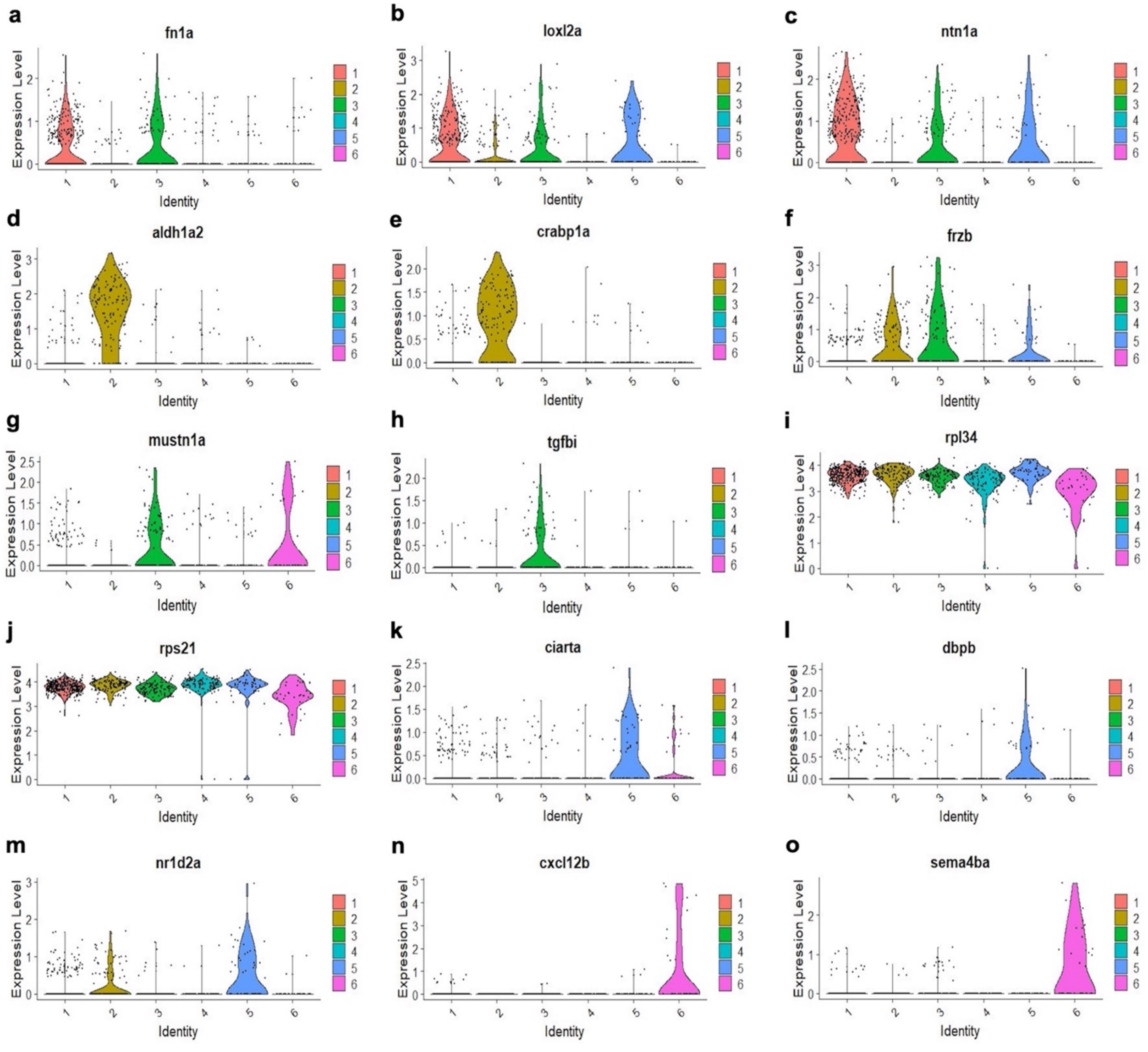

Supplementary Fig. 2: Violin plot comparing the expression of *fn1a (***a**), *loxl2a* (**b**), *ntn1a* (**c**), *aldh1a2* (**d**), *crabp1a* (**e**), *frzb* (**f**), *mustn1a* (**g**), *tgfbi* (**h**), *rpl34* (**i**), *rps21* (**j**), *ciarta* (**k**), *dbpb* (**l**), *nr1d2a* (**m**), *cxcl12b* (**n**), and *sema4ba* (**o**), in *tcf21*$^{+}$ cell clusters from juvenile hearts.

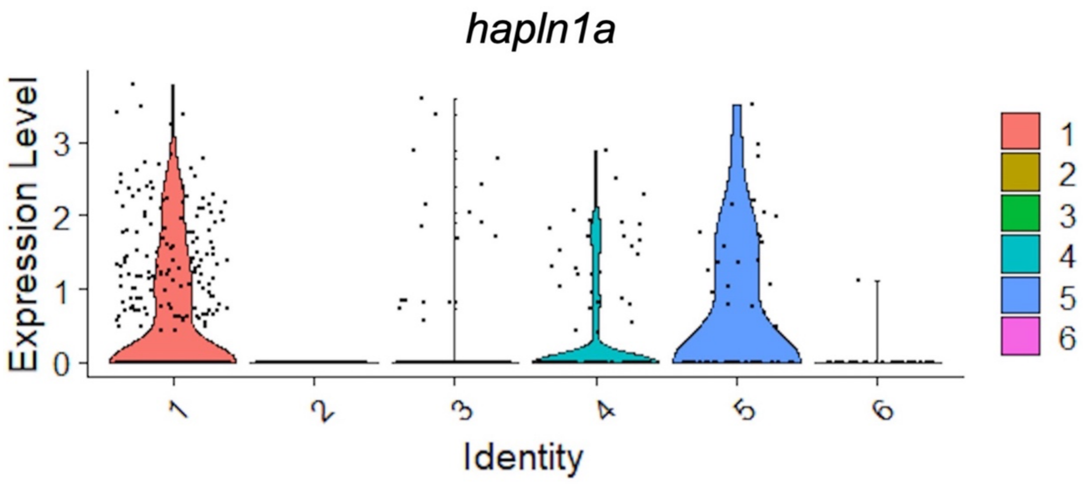

Supplementary Fig. 3: Violin plot comparing the expression of *hapln1a* in *tcf21*$^{+}$ cell clusters from juvenile hearts. *hapln1a* shows more expression in cluster 1 when compared with its expression in other clusters.

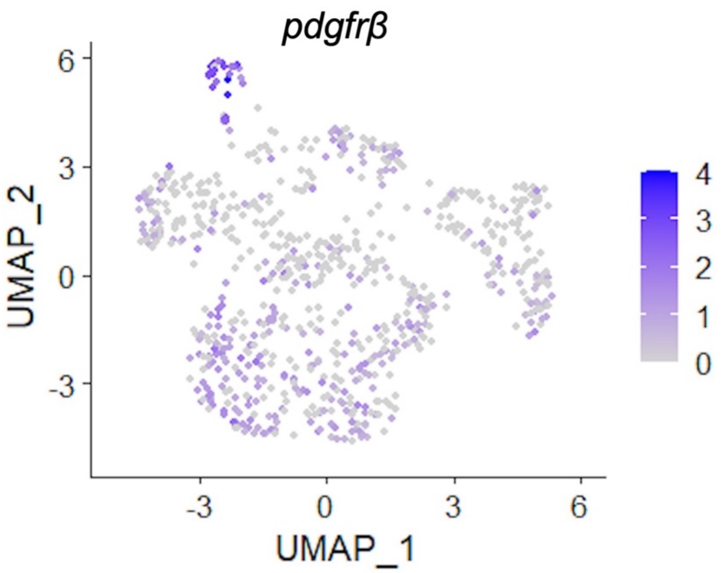

Supplementary Fig. 4: Feature plot of perivascular cell marker *pdgfrb* expression in *tcf21*$^{+}$ cell clusters at the juvenile stage.

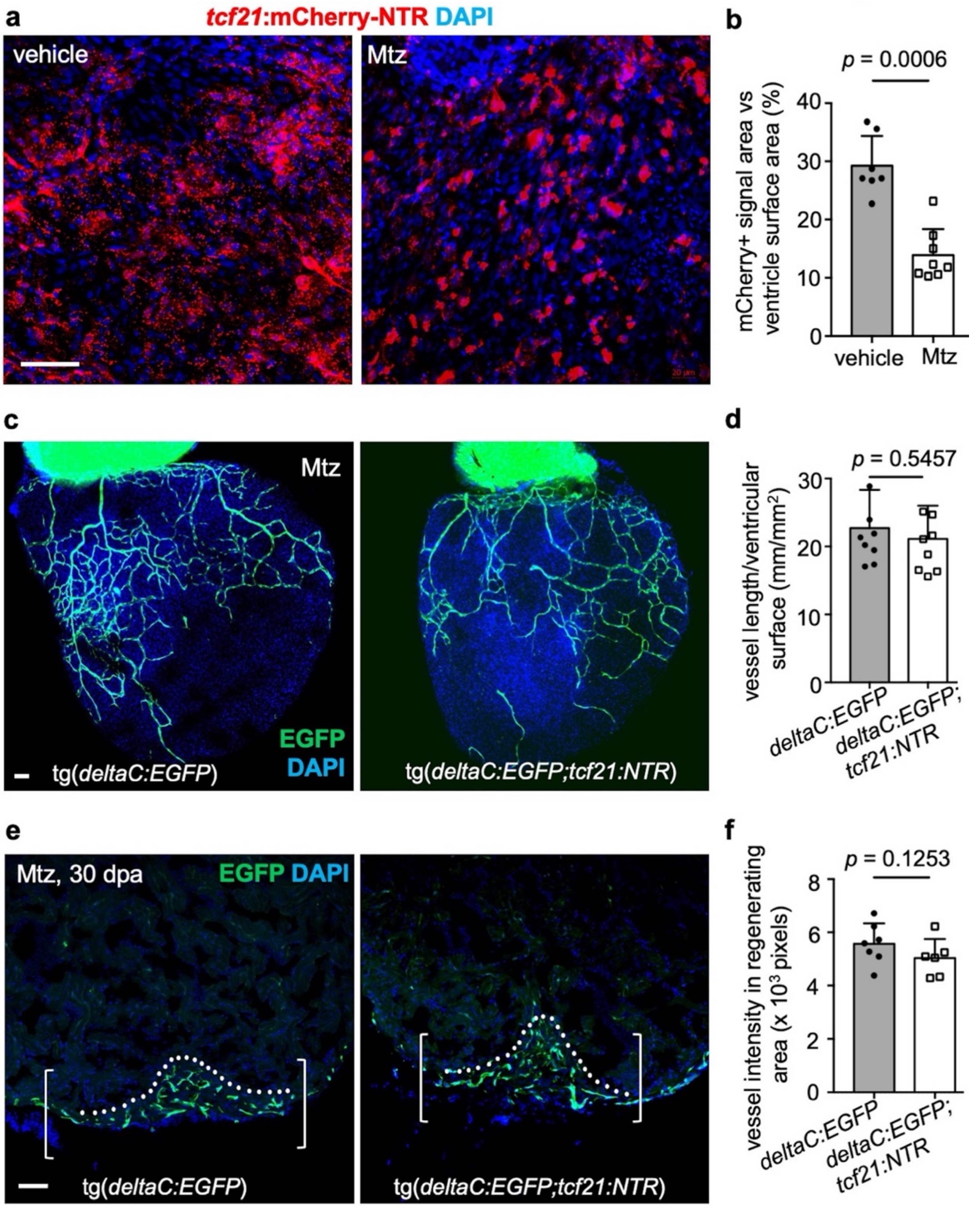

Supplementary Fig. 5. **Coronary vessel formation after depletion of around 50% of *tcf21*⁺ cells.** (**a**) Juvenile *tcf21:mCherry-NTR* animals were incubated with Mtz or vehicle for 12 hours, and hearts were collected by random sampling at 2 days post treatment. The ventricles with Mtz treatment showed obvious reduction of mCherry signals,

compared with vehicle controls. Scale bar, 50 μm. (**b**) Quantification of the percentage of mCherry^{+} pixels on the ventricular surface from experiments in (**a**). Total 7 vehicle- and 7 Mtz-treated hearts were used. Mann-Whitney Rank Sum test (Two-sided). Data are presented as mean values ± SD. Source data are provided as a Source Data file. The experiment was repeated once with similar results. (**c**) Whole-mount view of the ventricular surface from *in vivo* juvenile *deltaC:EGFP;tcf21:NTR* (n = 9) and *deltaC:EGFP* (n = 9) clutchmates that were treated with 10mM Mtz for 12 hours, and then the hearts were extracted at day 10 and analyzed. Scale bars, 50 μm. (**d**) Quantification of pixels of EGFP^{+} signals versus the whole ventricular area in experiment (**c**). Mann-Whitney Rank Sum test (Two-sided). Data are presented as mean values ± SD. Source data are provided as a Source Data file. The experiment was repeated once with similar results. (**e**) Section views of coronary vessels in the wounded area in adult injured *deltaC:EGFP;tcf21:NTR* animals and *deltaC:EGFP* siblings with Mtz treatment, by 30 dpa. Brackets represent the regenerated area. Scale bar, 50 μm. (**f**) Quantification of EGFP^{+} coronary cell pixels in the wound from (**e**). n = 6 *deltaC:EGFP* and *n* = 7 *deltaC:EGFP;tcf21:NTR* animals were used. Mann-Whitney Rank Sum test (Two-sided). Data are presented as mean values ± SD. Source data are provided as a Source Data file. The experiment was repeated once with similar results. Scale bars, 50 μm. The depletion of ~50% of *tcf21*$^{+}$ cells didn't show a significant difference in coronary vessel formation during morphogenesis and regeneration, compared with controls. These observations indicate that *hapln1a*$^{+}$ cell ablation has severe effects on coronary growth, compared with 50% *tcf21*$^{+}$ cell depletion.

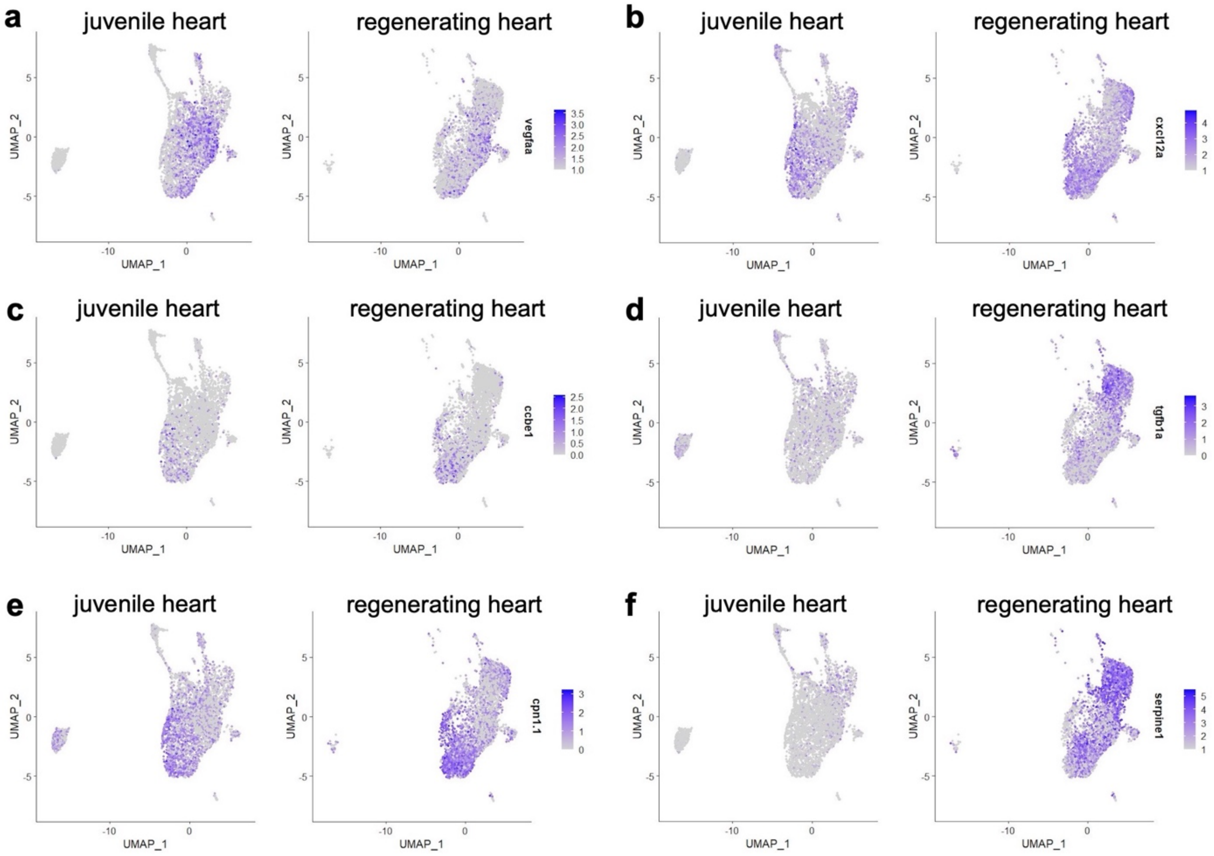

Supplementary Fig. 6: Feature plots of the expression of *vegfaa* (**a**), *cxcl12a* (**b**), *ccbe1* (**c**), *tgfb1a* (**d**), *cpn1.1* (**e**), *and serpine1* (**f**) in *hapln1a*$^{+}$ cell clusters of juvenile and regenerating hearts.

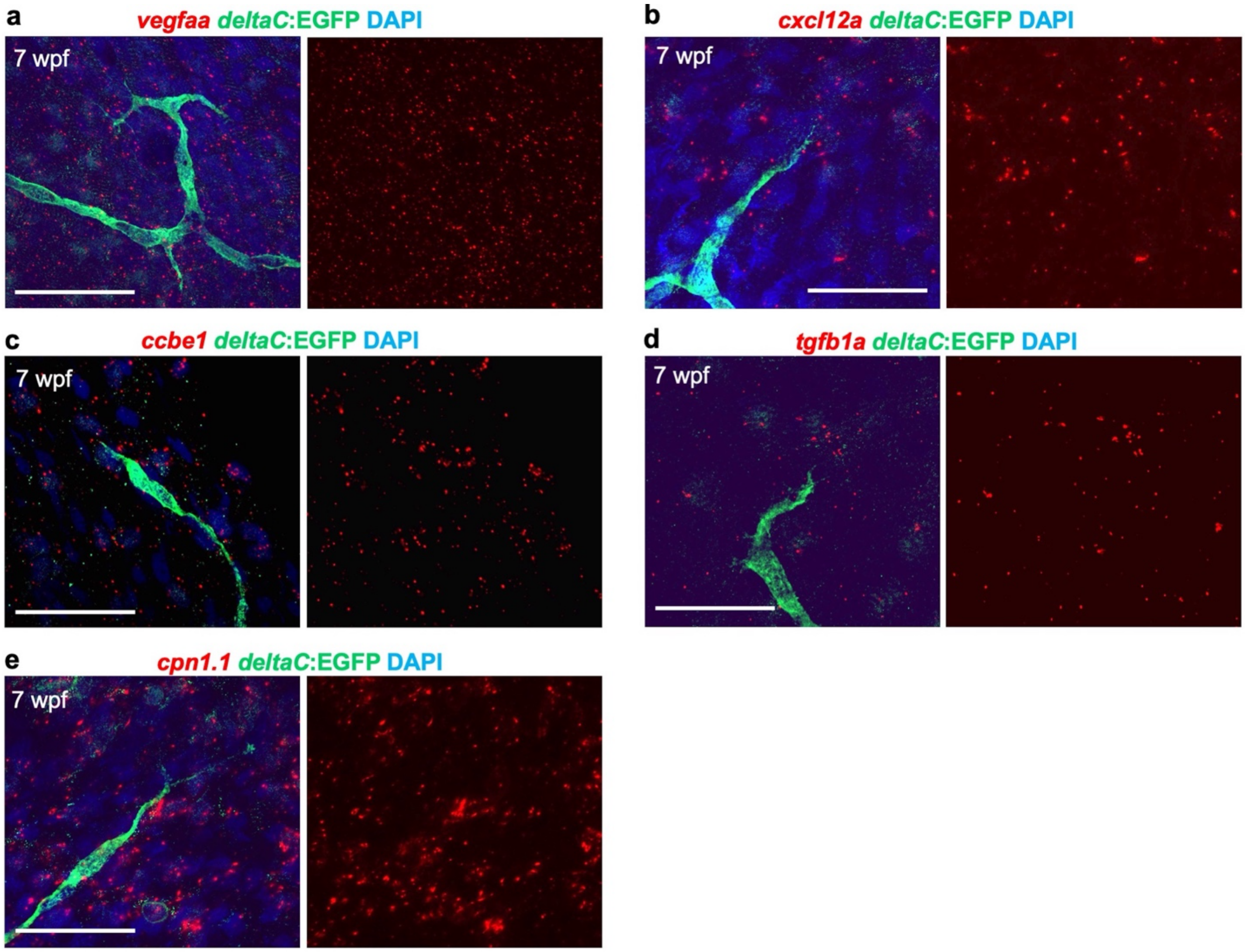

Supplementary Fig. 7: Visualization of coronary vessels and *in situ* signals of anti-sense RNA of *vegfaa* (**a**), *cxcl12a* (**b**), *ccbe1* (**c**), *tgfb1a* (**d**), and *cpn1.1* (**e**) in whole-mount view of the ventricular surface of juvenile *deltaC:EGFP* animals. n = 6-8. Scale bar, 50 µm.

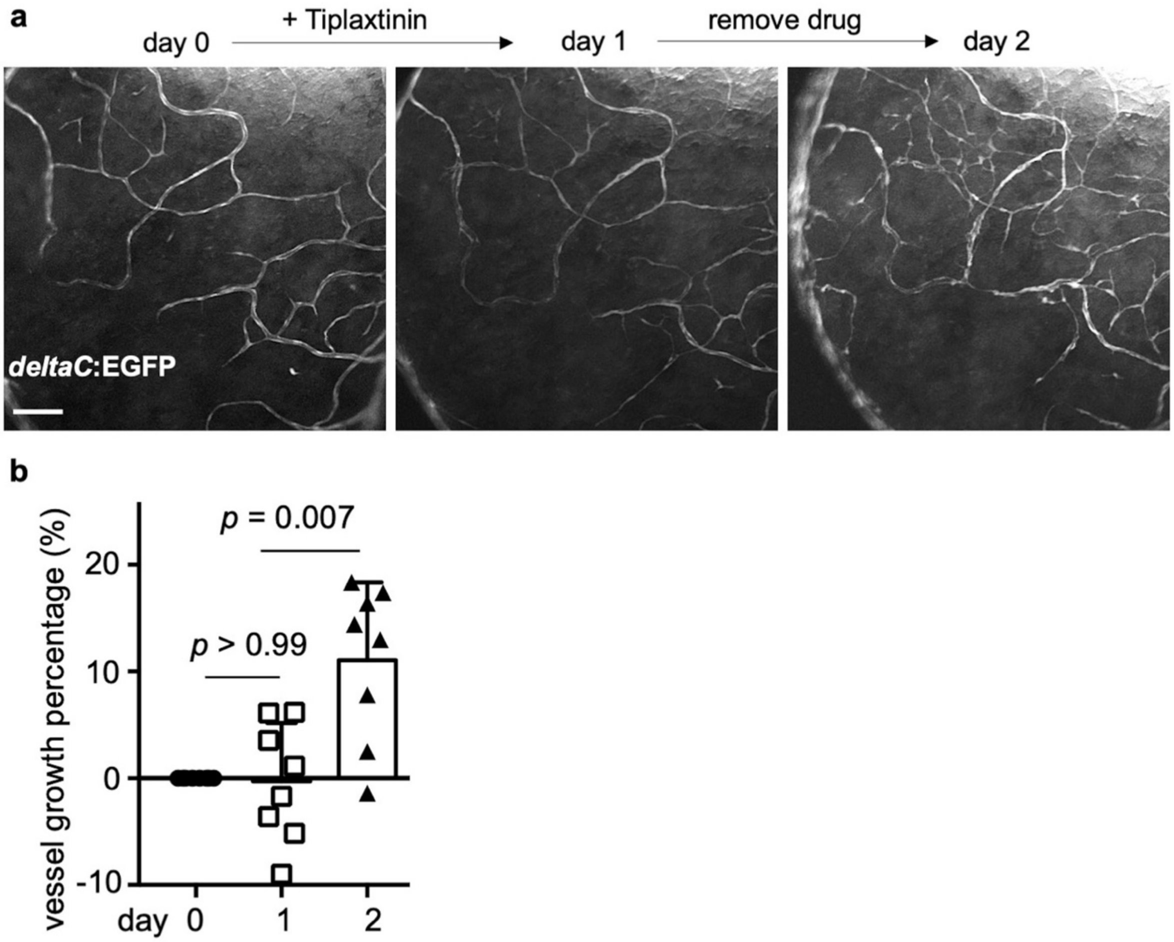

Supplementary Fig. 8: Serpine1 pathway activity controls coronary growth. (**a**) *deltaC:EGFP* hearts were extracted and incubated with Tiplaxtinin for 24 hours, then removed and cultured for another 24 hours. Scale bars, 50 μm. (**b**) Quantification of $EGFP^+$ vessel growth from experiments in (**a**). 8 animals were used. For day 1, a slight reduction (0.29%) was observed; for day 2, a significant increase of vessel growth (11.05%) was observed. Mann-Whitney Rank Sum test (Two-sided). Data are presented as mean values ± SD. Source data are provided as a Source Data file. The experiment was repeated once with similar results.
