# Supplementary material for: hapln1a+ cells guide coronary growth during heart morphogenesis and regeneration
Source: Nat Commun. 2023 Jun 13;14:3505. doi: 10.1038/s41467-023-39323-6 (PMC10264374; doi:10.1038/s41467-023-39323-6)
Supplement: Supplementary file 3 — Reporting Summary [file 41467_2023_39323_MOESM3_ESM.pdf]

nature portfolio

Corresponding author(s): Wang, Jinhu
Last updated by author(s): May 29, 2023

Reporting Summary

Nature Portfolio wishes to improve the reproducibility of the work that we publish. This form provides structure for consistency and transparency in reporting. For further information on Nature Portfolio policies, see our Editorial Policies and the Editorial Policy Checklist.

Statistics

For all statistical analyses, confirm that the following items are present in the figure legend, table legend, main text, or Methods section.

n/a	Confirmed	
☐	☒	The exact sample size (*n*) for each experimental group/condition, given as a discrete number and unit of measurement
☐	☒	A statement on whether measurements were taken from distinct samples or whether the same sample was measured repeatedly
☐	☒	The statistical test(s) used AND whether they are one- or two-sided<br>*Only common tests should be described solely by name; describe more complex techniques in the Methods section.*
☒	☐	A description of all covariates tested
☐	☒	A description of any assumptions or corrections, such as tests of normality and adjustment for multiple comparisons
☐	☒	A full description of the statistical parameters including central tendency (e.g. means) or other basic estimates (e.g. regression coefficient) AND variation (e.g. standard deviation) or associated estimates of uncertainty (e.g. confidence intervals)
☐	☒	For null hypothesis testing, the test statistic (e.g. *F*, *t*, *r*) with confidence intervals, effect sizes, degrees of freedom and *P* value noted<br>*Give P values as exact values whenever suitable.*
☒	☐	For Bayesian analysis, information on the choice of priors and Markov chain Monte Carlo settings
☒	☐	For hierarchical and complex designs, identification of the appropriate level for tests and full reporting of outcomes
☒	☐	Estimates of effect sizes (e.g. Cohen's *d*, Pearson's *r*), indicating how they were calculated

Our web collection on statistics for biologists contains articles on many of the points above.

Software and code

Policy information about availability of computer code

Data collection	Software used to data collection and analyses, and obtain single cell data are described fully in the Methods: ImageJ V1.53, Zeiss ZEN V3.7, FACSDiva v9.0, Cell Ranger Single-Cell Software V3.0.2.
Data analysis	Code used for single cell RNA-seq data analysis is available on a GitHub repository at https://github.com/jishengsun/tcf21_hapin1a_scRNAseJ

For manuscripts utilizing custom algorithms or software that are central to the research but not yet described in published literature, software must be made available to editors and reviewers. We strongly encourage code deposition in a community repository (e.g. GitHub). See the Nature Portfolio guidelines for submitting code & software for further information.

Data

Policy information about availability of data

All manuscripts must include a data availability statement. This statement should provide the following information, where applicable:

- Accession codes, unique identifiers, or web links for publicly available datasets
- A description of any restrictions on data availability
- For clinical datasets or third party data, please ensure that the statement adheres to our policy

The scRNA-seq dataset has been deposited in the National Center for Biotechnology Information Gene Expression Omnibus (GEO), with the GEO accession number GSE216649 which is publicly accessible. All other relevant data supporting the key findings of this study are available within the article and its Supplementary Information files or from the corresponding author upon reasonable request. Source data are provided with this paper.

Research involving human participants, their data, or biological material

Policy information about studies with human participants or human data. See also policy information about sex, gender (identity/presentation), and sexual orientation and race, ethnicity and racism.

Reporting on sex and gender	N/A
Reporting on race, ethnicity, or other socially relevant groupings	N/A
Population characteristics	N/A
Recruitment	N/A
Ethics oversight	N/A

Note that full information on the approval of the study protocol must also be provided in the manuscript.

Field-specific reporting

Please select the one below that is the best fit for your research. If you are not sure, read the appropriate sections before making your selection.

☒ Life sciences ☐ Behavioural & social sciences ☐ Ecological, evolutionary & environmental sciences

For a reference copy of the document with all sections, see nature.com/documents/nr-reporting-summary-flat.pdf

Life sciences study design

All studies must disclose on these points even when the disclosure is negative.

Sample size	Sample sizes were limited by practical issues (e.g. Labour intensity for surgery, financial constrains for profiling).
Data exclusions	To ensure at least a 95% cell viability following the entire procedure, we gated viable cells by negative SYTOXTM Red fluorescence and examined cell viability after cell sorting.
Replication	Experiments were repeated at least once with similar results.
Randomization	Animals in experimental groups were randomly assigned.
Blinding	Surgery, quantification and analyses were performed prior to indicating fish transgenic types and control animals.

Behavioural & social sciences study design

All studies must disclose on these points even when the disclosure is negative.

Study description	N/A
Research sample	N/A
Sampling strategy	N/A
Data collection	N/A
Timing	N/A
Data exclusions	N/A
Non-participation	N/A
Randomization	N/A

Ecological, evolutionary & environmental sciences study design

All studies must disclose on these points even when the disclosure is negative.

Study description	N/A
Research sample	N/A
Sampling strategy	N/A
Data collection	N/A
Timing and spatial scale	N/A
Data exclusions	N/A
Reproducibility	N/A
Randomization	N/A
Blinding	N/A

Did the study involve field work? ☐ Yes ☒ No

Field work, collection and transport

Field conditions	N/A
Location	N/A
Access & import/export	N/A
Disturbance	N/A

Reporting for specific materials, systems and methods

We require information from authors about some types of materials, experimental systems and methods used in many studies. Here, indicate whether each material, system or method listed is relevant to your study. If you are not sure if a list item applies to your research, read the appropriate section before selecting a response.

Materials & experimental systems

n/a	Involved in the study	
☐	☒	Antibodies
☒	☐	Eukaryotic cell lines
☒	☐	Palaeontology and archaeology
☐	☒	Animals and other organisms
☒	☐	Clinical data
☒	☐	Dual use research of concern
☒	☐	Plants

Methods

n/a	Involved in the study	
☒	☐	ChIP-seq
☐	☒	Flow cytometry
☒	☐	MRI-based neuroimaging

Antibodies

Antibodies used	Primary antibodies used in this study include IB4 (Vector labs, DL-1208-5, 1:100), HuC/HuD (Invitrogen, A21271, 1:100), AcT (Sigma-Aldrich, T6793-.2ML, 1:100), and GFP (Ayes Labs, GFP-1020, 1:200). Alexa Fluor secondary antibodies used include 488 (goat anti-Chicken, A-11039, 1:200) and 594 (goat anti-mouse, A-11005, 1:200).
Validation	The IB4 antibody has been used in 330 papers from pubmed; the huC/HuD has been used in 40 publications from pubmed; The AcT has been used in more than 50 publications from pubmed; the anti-GFP has been used in 15 publications from Zfin. All secondary antibodies used were more than 500 publications from PubMed.

Eukaryotic cell lines

Policy information about cell lines and Sex and Gender in Research

Cell line source(s)	N/A
Authentication	N/A
Mycoplasma contamination	N/A
Commonly misidentified lines (See ICLAC register)	N/A

Palaeontology and Archaeology

Specimen provenance	N/A
Specimen deposition	N/A
Dating methods	N/A

☐ Tick this box to confirm that the raw and calibrated dates are available in the paper or in Supplementary Information.

Ethics oversight	N/A

Note that full information on the approval of the study protocol must also be provided in the manuscript.

Animals and other research organisms

Policy information about studies involving animals; ARRIVE guidelines recommended for reporting animal research, and Sex and Gender in Research

Laboratory animals	zebrafish (Danio rerio), EK, AB strains, Tg(hapln1a:mCherry-NTR)em14Tg, Tg(tcf21:nucEGFP)pd41Tg , Tg(hapln1a:EGFP)pd338Tg, Tg(hapln1a:mCherry-NTR)em14Tg, Tg(deltaC:EGFP)em11Tg, Tg(deltaC:mCherry)em11Tg. All transgenic strains were analyzed as hemizygotes. Zebrafish were used at the age of 6-7 weeks with a length between 1.5 – 1.8 cm (juvenile), or 4-10 months with a length of at least 3 cm (adult).
Wild animals	No wild animals were used.
Reporting on sex	All animals were used with male to female at 1:1 when sex determination has occurred.
Field-collected samples	No samples collected from field.
Ethics oversight	All animal procedures were performed in accordance with Emory University guidelines.

Note that full information on the approval of the study protocol must also be provided in the manuscript.

Clinical data

Policy information about clinical studies

All manuscripts should comply with the ICMJE guidelines for publication of clinical research and a completed CONSORT checklist must be included with all submissions.

Clinical trial registration	N/A
Study protocol	N/A
Data collection	N/A
Outcomes	N/A

Dual use research of concern

Policy information about dual use research of concern

Hazards

Could the accidental, deliberate or reckless misuse of agents or technologies generated in the work, or the application of information presented in the manuscript, pose a threat to:

No	Yes	
☒	☐	Public health
☒	☐	National security
☒	☐	Crops and/or livestock
☒	☐	Ecosystems
☒	☐	Any other significant area

Experiments of concern

Does the work involve any of these experiments of concern:

No	Yes	
☒	☐	Demonstrate how to render a vaccine ineffective
☒	☐	Confer resistance to therapeutically useful antibiotics or antiviral agents
☒	☐	Enhance the virulence of a pathogen or render a nonpathogen virulent
☒	☐	Increase transmissibility of a pathogen
☒	☐	Alter the host range of a pathogen
☒	☐	Enable evasion of diagnostic/detection modalities
☒	☐	Enable the weaponization of a biological agent or toxin
☒	☐	Any other potentially harmful combination of experiments and agents

Plants

Seed stocks	N/A
Novel plant genotypes	N/A
Authentication	N/A

ChIP-seq

Data deposition

☐ Confirm that both raw and final processed data have been deposited in a public database such as GEO.

☐ Confirm that you have deposited or provided access to graph files (e.g. BED files) for the called peaks.

Data access links<br>*May remain private before publication.*	N/A
Files in database submission	N/A
Genome browser session<br>(e.g. UCSC)	N/A

Methodology

Replicates	N/A
Sequencing depth	N/A
Antibodies	N/A
Peak calling parameters	N/A
Data quality	N/A
Software	N/A

Flow Cytometry

Plots

Confirm that:

☒ The axis labels state the marker and fluorochrome used (e.g. CD4-FITC).

☒ The axis scales are clearly visible. Include numbers along axes only for bottom left plot of group (a 'group' is an analysis of identical markers).

☒ All plots are contour plots with outliers or pseudocolor plots.

☒ A numerical value for number of cells or percentage (with statistics) is provided.

Methodology

Sample preparation	To prepare tcf21+ and hapIn1a+ cells for single-cell RNA-sequencing analyses: 80 tcf2l:nucEGFP and 120 hapln1a:EGFP fish were raised to the juvenile stage, and 60 hapln1a:EGFP fish were raised to the adult stage. Juvenile hearts were collected at 7 wpf (1.6 —1.8 cm length). The hearts of adult fish at 6 months old (3.5 — 4 cm length) were extracted at 7 days post amputation (dpa) and the wounded apex region was collected. The heart samples were digested with 0.26 U/mL LiberaseTM Thermolysin Medium (TM). Dissociated cells were spun down and live EGFP+ cells were sorted by flow cytometry. To ensure at least a 95% cell viability following the entire procedure, we gated viablecells by negative SYTOXTM Red fluorescence and examined cell viability after cell sorting. Isolated cells were sent to the Emory Integrated Genomics Core (EIGC) center for 10x single-cell RNA-sequencing.
Instrument	BD FACSAria III cell sorter
Software	BD FACSDiva V9.0
Cell population abundance	9-10% of population 1
Gating strategy	Total events were visualized with a FSC-A Vs SSC scatter plot from which the main Popultion 1 (P1) was gated to discard cell debris.Exclusion of double event was performed using a FSC-A Vs FSC-W scatter plot from which population 2 (P2) was gated. GFP and DAPI was assessed using a two parameter scatter plot. These information has been mentioned in Supplementary information_Supplementary Figure 1_Legend.

☒ Tick this box to confirm that a figure exemplifying the gating strategy is provided in the Supplementary Information.

Magnetic resonance imaging

Experimental design

Design type	N/A
Design specifications	N/A
Behavioral performance measures	N/A

Acquisition

Imaging type(s)	N/A
Field strength	N/A
Sequence & imaging parameters	N/A
Area of acquisition	N/A

Diffusion MRI ☐ Used ☐ Not used

Preprocessing

Preprocessing software	N/A
Normalization	N/A
Normalization template	N/A
Noise and artifact removal	N/A

Volume censoring | N/A

Statistical modeling & inference

Model type and settings | N/A

Effect(s) tested | N/A

Specify type of analysis: ☐ Whole brain ☐ ROI-based ☐ Both

Statistic type for inference | N/A

(See Eklund et al. 2016)

Correction | N/A

Models & analysis

n/a	Involved in the study
☒	☐ Functional and/or effective connectivity
☒	☐ Graph analysis
☒	☐ Multivariate modeling or predictive analysis

Functional and/or effective connectivity | N/A

Graph analysis | N/A

Multivariate modeling and predictive analysis | N/A
